# Imported Dengue Fever in Milan, Italy: A Seven-Year Retrospective Study

**DOI:** 10.3390/idr17050113

**Published:** 2025-09-12

**Authors:** Margherita Eleonora Pieruzzi, Davide Mileto, Alessandra Helen Behring, Stefania Caronni, Alessandro Mancon, Luigi Vezzosi, Alberto Rizzo, Andrea Poloni, Andrea Gori, Andrea Giacomelli, Spinello Antinori

**Affiliations:** 1 Department of Biomedical and Clinical Sciences, Università degli Studi di Milano, 20157 Milano, Italy; margherita.pieruzzi@unimi.it (M.E.P.); alessandra.behring@unimi.it (A.H.B.); andrea.gori@unimi.it (A.G.); andrea.giacomelli@unimi.it (A.G.); 2 III Division of Infectious Diseases, Luigi Sacco Hospital, Azienda Socio Sanitaria Territoriale (ASST) Fatebenefratelli (FBF) Sacco, 20157 Milano, Italy; 3 Laboratory of Clinical Microbiology, Virology and Bioemergencies, Luigi Sacco Hospital, Azienda Socio Sanitaria Territoriale (ASST) Fatebenefratelli (FBF) Sacco, 20157 Milano, Italy; davide.mileto@asst-fbf-sacco.it (D.M.); mancon.alessandro@asst-fbf-sacco.it (A.M.); rizzo.alberto@asst-fbf-sacco.it (A.R.); 4 Department of Infectious Diseases, Azienda Socio Sanitaria Territoriale (ASST) Fatebenefratelli (FBF) Sacco, 20157 Milano, Italy; stefania.caronni@asst-fbf-sacco.it (S.C.); andrea.poloni@asst-fbf-sacco.it (A.P.); 5 Directorate General for Health, Lombardy Region, 20124 Milano, Italy; luigi_vezzosi@regionelombardia.it; 6 Multidisciplinary Research in Health Science (MACH), Università degli Studi di Milano, 20157 Milano, Italy

**Keywords:** dengue fever, imported, Italy, travelers, warning signs

## Abstract

Background: Dengue fever is an arboviral infection transmitted by *Aedes* mosquitoes that has recently become a public health concern also in Europe, causing many outbreaks of autochthonous cases. Methods: We retrospectively retrieved dengue cases in returning travelers from tropical areas diagnosed at the Luigi Sacco Hospital between January 2018 and December 2024. All patients with positive serology for DENV (IgM alone or IgM and IgG or neutralizing antibodies detection) and/or positive real-time polymerase chain reaction (RT-PCR) for DENV RNA on plasma and urine were considered. Analyses were descriptive. Results: This analysis included 159 travelers with confirmed (*n* = 138; 86.8%) or probable (*n* = 21; 13.2%) dengue fever. The median age was 38 years (interquartile range [IQR] 30–50); 87 (54.7%) were females. The median time between symptom onset and seeking medical attention was four days (IQR 3–6); 29.6% required hospitalization, with a median stay of four days (IQR 0–5). The most common symptoms included fever (100%), myalgia (52.2%), and headache (49.1%). Laboratory findings revealed thrombocytopenia (53.8%), leukopenia (64.1%), elevated ALT (51.9%), and LDH (60.2%). Among the confirmed cases, 128 (92.8%) were diagnosed with RT-PCR. Serotypes 1 and 2 were the most prevalent (28.9% and 27.3%, respectively). Most cases were classified as dengue without warning signs (150, 94.3%), eight cases (5.0%) as dengue with warning signs, and one as severe dengue. Conclusions: Dengue fever is an important cause of fever among travelers returning to Italy from endemic areas. Although severe dengue is rare among travelers, further prospective studies need to address this issue. Diagnosis should be pursued by using molecular tools because of cross-reactivity with other arboviruses.

## 1. Background

The viral species *Orthoflavivirus denguei*, viral name Dengue virus (DENV), is responsible for dengue fever, an arboviral infection transmitted by *Aedes* mosquitoes that is endemic in more than 100 countries worldwide [1]. Dengue virus is an enveloped single-stranded RNA virus belonging to the Flaviviridae family, genus *Orthoflavivirus*, classified into four serotypes (DENV-1, DENV-2, DENV-3, and DENV-4) sharing approximately 65% of the genome [2]. Climate change, population growth, urbanization, and human mobility are expected to exacerbate the global dengue burden by increasing the risk in endemic areas but also by introducing the virus into new areas. International travel is considered a major driver in the spreading of DENV with the introduction of the virus in geographic locations where suitable vectors are present, such as Italy, France, and Spain, where *Aedes albopictus* is widespread [3,4,5]. In Italy, since 2020, three autochthonous outbreaks of dengue fever have occurred, and in 2024, 213 cases were reported from six Italian regions [6,7,8,9]. The 2020 outbreak in Northern Italy and the 2023 outbreak in Lombardy were caused by DENV-1, whereas the recent 2024 outbreak in Central Italy was due to DENV-2. Additionally, a few autochthonous cases of dengue caused by DENV-3 have been reported.

The objective of this study was to describe epidemiological, virological, and clinical characteristics among returned international travelers diagnosed with dengue at Luigi Sacco University Hospital in Milan, Italy, over a seven-year period.

## 2. Materials and Methods

### 2.1. Study Design, Setting and Participants

This was a retrospective monocentric study conducted at Luigi Sacco Hospital, Azienda Socio Sanitaria Territoriale (ASST) Fatebenefratelli Sacco, Milan, Italy. L. Sacco University Hospital is a public Italian center of excellence for research, diagnosis, and clinical care of infectious diseases. The availability of high containment facilities makes the hospital a regional and national referral for diagnosis and management of Risk 3 and 4 biological agents and bioterrorism. Our laboratory is one of the only two public reference labs of the Lombardy Region authorized by the Ministry of Health for arbovirus diagnosis.

Individuals aged above 18 years who presented to the emergency department or an outpatient clinic of our ASST and had a positive serology for DENV and/or positive real time polymerase chain reaction (RT-PCR) for DENV RNA on plasma and/or urine between January 2018 and December 2024 were included in this present analysis.

### 2.2. Data Collection, Variables, and Classification

All diagnostic procedures were performed at the Laboratory of Clinical Microbiology, Virology, and Bioemergencies, ASST Fatebenefratelli Sacco (Milan, Italy). Electronic health records of the laboratory were queried to identify individuals with a positive serology for DENV antibodies (IgM alone or IgM and IgG or neutralizing antibodies detection) and/or positive RT-PCR for DENV RNA on plasma and/or urine. The corresponding clinical health records were assessed to retrieve the following information: age, gender, nationality, travel destination, reason of travel, travel period (date of beginning and end of travel, travel duration), date of symptom onset, date of presentation in the emergency department, time between arrival in Italy and symptoms’ onset, time between symptom onset and presentation in the emergency department, previous contact with medical care before hospital evaluation, antibiotics use before hospital evaluation, signs and symptoms at presentation, hematologic and biochemical tests’ results (complete blood count, prothrombin time, creatinine, lactate dehydrogenase, transaminases, blood bilirubin, gamma glutamyl transpeptidase, alkaline phosphatase), radiological exams, requirement of hospitalization and median stay, vaccination for dengue.

Travel destinations were grouped under six World Health Organization (WHO) regions (Americas, African, Southeast Asia, Eastern Mediterranean, Western Pacific, and European) [10].

Leukopenia was defined as a white blood cell count ≤ 4000 cells/µL, and thrombocytopenia as a platelet count ≤ 150,000 cells/µL. Negative CRP was defined as <5 mg/L [11].

Dengue cases were classified according to WHO 2009 guidelines as dengue without warning signs, dengue with warning signs, and severe dengue [12].

Luigi Sacco Hospital was affected by the COVID-19 pandemic beginning in February 2020, with recurrent surges of cases that required a large portion of the hospital beds to be dedicated to the management of infected patients until the end of 2021.

### 2.3. Diagnostic Methods

Real-time PCR for DENV RNA detection was used to test plasma and urine samples. From 2018 to 2022, the multiplex assay Clonit’ngo Zika, Dengue, Chikungunya (CLONIT srl, Milan, Italy) was used as a screening test, and DENV serotype was identified by the quantitative Dengue Serotype Virus kit (CLONIT srl, Milan, Italy), while in 2023 only the quantitative assay was used as a screening and serotyping method. From January 2024, all samples were simultaneously screened with home-made real-time PCR Pan-Orthovirus and RealStar Dengue RT-PCR kit 2.0 (altona Diagnostics, Hamburg, Germany) for viral serotyping identification. A Ct > 40 was set as a negative cut-off threshold for all RT-PCR methods. The specific DENV IgM and IgG were assayed in serum samples by means of chemiluminescent assay (Dengue VirClia IgM monotest and Dengue VirClia IgG monotest, VirCell Microbiologists, Granada, Spain). Results were expressed as an index and were given as positive when the index was higher than 1.1. Serum virus neutralization assay (SVN), the laboratory gold-standard test for detection and quantitation of specific neutralizing antibodies against DENV, was conducted using DENV serotype 2 on serum samples to confirm the IgM and/or IgG detected by chemiluminescent screening. Because of the serum cross-reactivities among Orthoflavivirus, each sample was tested simultaneously for dengue virus, West Nile virus, Zika virus, Chikungunya, and Usutu virus. A cut-off of >1:10 was used for positive results. Neutralization assay details were previously reported [13].

Confirmed cases of dengue infection were defined as follows [14]:

- DENV RNA detection by RT-PCR on plasma and/or urine samples.
- Dengue-specific IgM antibodies in a single serum sample confirmed by neutralization assay.
- Seroconversion or four-fold IgG titer increases in paired serum samples.

Probable cases were defined by positivity of dengue-specific IgM antibodies in a single serum sample of patients with clinical signs and symptoms compatible with dengue fever and/or epidemiological link.

### 2.4. Stastistical Analysis

A descriptive statistical analysis was carried out. Continuous variables were described as median and interquartile range (IQR). Discrete variables were described as absolute numbers and percentages.

## 3. Results

### 3.1. Characteristics of the Study Population

A total of 159 dengue cases were identified, comprising 138 confirmed (86.8%) and 21 probable (13.2%) cases. No autochthonous cases were detected, and coinfections with other arboviruses were identified. Demographic characteristics and travel history are summarized in Table 1.

The median age was 38 years (interquartile range [IQR] 30–50), and 54.7% were females. The median duration of travel was 17 days (IQR 12–22); 34 individuals had incomplete travel history. In total, 87 out of 159 patients (54.7%) were already symptomatic either during their stay abroad or on the day of return to Italy.

The median time from symptom onset to seeking medical care was four days (IQR 3–6).

Clinical features and laboratory findings are shown in Table 2.

Most cases were classified as dengue without warning signs (*n* = 150; 94.3%), while eight patients (5.0%) were diagnosed with dengue with warning signs. All patients in this group experienced bleeding, predominantly mucosal (e.g., epistaxis, gingival bleeding, vaginal bleeding), and in one case, hematemesis. One patient (0.6%) was classified as having severe dengue due to anemia, respiratory distress, and pleural effusion (Table 1).

**Figure 1:**
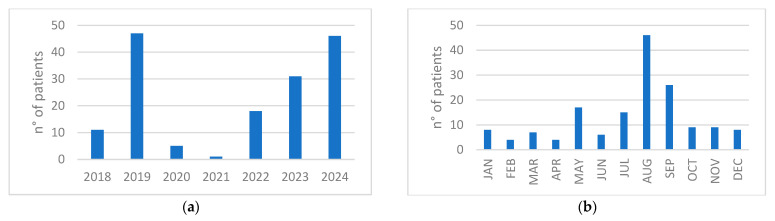
(**a**) Distribution of cases per year; (**b**) Distribution of cumulative number of patients per month during the years 2018–2024.

The distribution of cases per year showed a reduced amount of diagnoses in 2020–2021, corresponding with the SARS-CoV-2 pandemic and associated global travel restrictions, followed by a progressive absolute increase in 2022, 2023, and 2024 (Figure 1a). Most dengue cases were observed during the months of August and September, with the monthly distribution throughout the year shown in Figure 1b.

**Figure 2:**
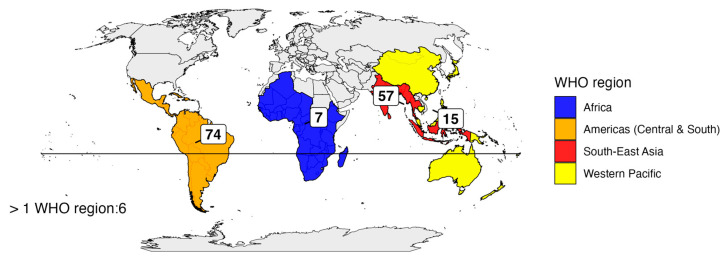
Distribution of travelers in the five WHO regions.

The primary reason for travel was tourism (*n* = 101; 63.5%), followed by visiting friends and relatives (VFR) in 30 cases (18.9%) and work or volunteering in 10 cases (6.3%). For 18 patients (11.3%), the reason for travel was not available. The most frequently reported travel destination was Central and South America (*n* = 74, 46.5%), followed by Southeast Asia (*n* = 57, 35.8%) (Supplementary Table S1). Fifteen patients (9.4%) traveled to the Western Pacific region, seven (4.4%) to Africa, and six patients (3.8%) had visited more than one geographic area (Figure 2).

We assessed dengue vaccination status for patients who traveled during 2023–2024. Three patients, all classified as probable dengue cases, had received the dengue vaccine approved in Italy (Qdenga^®^) prior to travel; only one of them had completed the full two-dose regimen.

### 3.2. Laboratory Analysis and Classification

Real-time polymerase chain reaction (RT-PCR) on plasma samples was performed in 151 (95%) patients, with 118 testing positive (78.1%) (Table 3).

Urine RT-PCR was conducted in 125 (78.6%) patients, of whom 72 were positive (57.6%). Among patients (147, 92.5%) who underwent both tests, 62 (42.2%) were positive in both plasma and urine samples.

Overall, 121 patients underwent RT-PCR diagnostic testing within seven days of symptom onset, with 108 of them testing positive (89.3%). In a single patient, detectable DENV RNA in serum persisted for thirty-four days (22 October–24 November).

Dengue IgM antibodies were searched in 153 (96.2%) patients, with 109 (71.2%) positives, while IgG antibodies were assessed in 151 (95%) patients, of whom 66 (43.7%) tested positive (index range 1.1–10.2). Overall, 54 patients out of 153 (35.3%) who underwent both assays tested positive for both IgM and IgG antibodies (Table 3).

A total of 50 patients (43.9%) had both positive RNA and IgG antibodies, suggesting a probable secondary dengue infection. Among the 138 confirmed cases, 128 (92.8%) were diagnosed by RT-PCR: 56 (35.9%) on plasma only, 10 (7.8%) on urine only, and 62 (48.4%) on both. Of the remaining 10, 3 were confirmed through neutralization tests (titer range 1:20–1:80), and the others by seroconversion between acute and convalescent samples.

Among the 21 cases classified as probable dengue, 16 (76.2%) did not undergo follow-up serology after 15 days. Additionally, RT-PCR was not performed in 4 of these patients.

Dengue virus serotype was identified in 120 patients (92.3%), with serotypes DENV-1 (28.7%) and DENV-2 (27.1%) being the most prevalent (Table 4).

## 4. Discussion

We have analyzed dengue fever cases diagnosed during a seven-year period at the Department of Infectious Diseases of Luigi Sacco Hospital in Milan, Italy. Overall, 159 cases of dengue fever, 128 confirmed and 21 probable, were diagnosed at our center. As expected, we have observed a sharp decrease in imported dengue fever due to the COVID-19 pandemic during the years 2020–2021, but in 2024 the number of cases was again consistent with the peak observed in 2019 [15]. Considering the years 2018–2022, for whom data from the European Center for Disease Prevention and Control (ECDC) were available, in our center in Milan, 15.4% of all cases of dengue reported in Italy (82/531) were diagnosed during the same period [16].

The epidemiological characteristics of our population did not differ significantly from those reported by the latest analysis from the GeoSentinel network, a global network (tracking travel-related illness) with worldwide sites including Italy, except for a higher median age of our patients (38 vs. 33 years) and a shorter median travel duration (17 vs. 21 days) [17]. On the contrary, the most frequent regions of dengue exposure in our experience were Central and South America (46.5%), whereas dengue exposure in Southeast Asia was recorded only in 35.8% of subjects against 50.4% in the GeoSentinel analysis. This result probably reflects the preferred destinations of Italian travelers.

Patients typically experienced symptom onset shortly before or immediately after returning to Italy and sought medical attention at a specialized center within approximately four days.

Interestingly, nearly one-third of patients had already consulted a general practitioner or another emergency department prior to their arrival at our hospital, where the correct diagnosis was suspected and confirmed. Furthermore, around 20% were treated with unnecessary empiric antibiotic therapy. Both these facts emphasize the low awareness about dengue fever and other arboviruses among physicians outside the infectious diseases specialty. This is particularly worrisome in our country, where three outbreaks of autochthonous dengue fever were registered in 2020, 2023, and 2024, the last one with the highest number of cases ever reported in Europe [6,7,8,9].

In terms of clinical presentation, our findings are consistent with those of other studies, with fever, headache, and myalgia as the top three most frequently reported symptoms [17,18]. It is worth noting that dengue fever rarely presents itself as an isolated fever. Moreover, gastrointestinal symptoms were common among our patients, and particularly diarrhea was reported by nearly one-third of them, a finding observed also in a study of German travelers [19]. As far as rash, in our experience it was observed in about 38% of cases; that is a prevalence equal to what has been reported in a recent large autochthonous outbreak in Italy but slightly higher than in the GeoSentinel study (29%) [17]. However, in a systematic review of dengue in the United States (comprising also studies conducted in Puerto Rico), a rash was reported in 12–71% of patients in the different studies [20]. It is unknown if these wide differences might be influenced by infections by different serotypes.

Severe dengue was diagnosed in less than 1% of our cases, which is consistent with the frequency reported in the largest study so far published among travelers (27/5958, 0.4%) by the GeoSentinel network as well as in another study in Czech travelers (0.75%) [21,22]; however, only 5% of our patients had warning signs, which is consistent with the results of a systematic review and meta-analysis of dengue infection in Europe showing that Italy had the highest proportion of dengue without warning signs [23].

Half of our patients had thrombocytopenia, and nearly two-thirds presented leukopenia; that is a slightly lower frequency with respect to previous experience in our center [24]. Notably, 43% of patients at presentation had normal C-reactive protein (CRP) levels: this finding should strengthen the clinical suspicion of dengue, as it is a common finding in arboviral infections [25].

Diagnosing dengue in non-endemic areas poses several challenges [26]. Many existing studies rely primarily on serological testing, offering limited insight into the diagnostic accuracy of various tools [19,22,27]. Serology is limited by cross-reactivity with other flaviviruses, the need for paired testing to demonstrate seroconversion, and potential interference from prior vaccination for dengue fever and yellow fever [28,29,30]. In our cohort, combined RT-PCR was the most effective diagnostic tool, offering high specificity and early detection, with approximately 93% of dengue fever cases confirmed by such molecular methods either on plasma or urine. Notably, 78% had a positive PCR on plasma and 57% on urine, underscoring the need to test both these samples to maximize the possibility of achieving a definite diagnosis. This approach was particularly effective in our setting, as the population attending our center consists mainly of tourists who seek medical attention promptly after symptom onset, when they are still viremic.

Interestingly, 20 out of 128 (15.6%) RT-PCR-positive patients were found to be positive beyond seven days from symptom onset. This might be the consequence of the difficulties in accurately determining the onset of illness. However, we cannot exclude the possibility of some cases of prolonged viremia. This feature, combined with the increasing number of diagnosed cases, may contribute to the annual risk of autochthonous outbreaks of dengue fever in our country, as observed in 2024, when multiple regions were involved [31]. All four dengue serotypes were imported by travelers irrespective of which region they had visited, except for serotypes 1 and 4 not detected from Africa; however, the low number of people coming from Africa precludes any conclusion. Overall, the frequency of dengue serotypes was like that reported in the TropNet study by Neumayr et al. [32].

Only three patients in our cohort had received the dengue vaccine, of whom only one completed the two-dose schedule. Although no consideration can be given due to the very low number of vaccinated subjects, it is notable that all such patients were diagnosed as probable dengue. So far, the knowledge of efficacy of the dengue vaccination among travelers is very limited, and a recent study from Germany (probably the country with the highest number of vaccinated travelers) reported that in the period 2023–2024, 2.3% of notified cases had been vaccinated against dengue [33].

This study has several limitations. It is a retrospective, single-center study, and it includes the years affected by the SARS-CoV-2 pandemic, during which global travel patterns and disease reporting were significantly disrupted. Symptom data were subject to the discretion of the physician in charge, with a possible bias. Third, we could not genotype dengue serotypes. These factors may have influenced the representativeness and generalizability of our findings. Nevertheless, we acknowledge several strengths: it was conducted in Milan, the city of Northern Italy hosting the largest Infectious Diseases Department that is a reference center for the diagnosis of imported infectious diseases; moreover, our laboratory is one of the two reference regional microbiology laboratories authorized by the Italian Ministry of Health for the diagnosis of arboviruses.

## 5. Conclusions

A marked decline in dengue cases was observed in 2020 and 2021, the years most affected by the SARS-CoV-2 pandemic, followed by a rapid increase in cases since 2023. The return of a high number of travelers from dengue-endemic regions, particularly during the summer months when *Aedes albopictus* is abundant in Italy, raises the risk of local transmission. This underscores the need for heightened awareness of the disease to prevent further autochthonous outbreaks in our country.

## Figures and Tables

**Table 1:** Patients’ baseline characteristics and travel data.

	*N* = 159
Age, median (IQR)	38 (30–50)
Female sex, *n* (%)	87 (54.7)
Italian, *n* (%)	127 (79.9)
**Reason for travel, *n* (%)**	
Tourism	101 (63.5)
Visiting friends and relatives	30 (18.9)
Business/volunteer	10 (6.3)
Not available data	18 (11.3)
**WHO region, *n* (%)**	
America (Central and South)	74 (46.5)
Southeast Asia	57 (35.8)
Western Pacific	15 (9.4)
>1 WHO region	6 (3.8)
African	7 (4.4)
Days of travel, median (IQR) *	17 (12–22)
Days between symptom onset and arrival to Italy, median (IQR)	0 (−3–+3)
Days between symptom onset and medical care, median (IQR)	4 (3–6)
**Medical attention before emergency department, *n* (%)**	51 (32.1)
Other hospital’s ED	29 (18.2)
Family doctor	20 (12.6)
Both	2 (1.3)
Previous antibiotic exposures, *n* (%)	32 (20.1)

IQR, interquartile range; WHO, World Health Organization; ED, emergency department. * Data not available for 34 patients.

**Table 2:** Patients’ clinical features and laboratory findings.

	*N* = 159
**Symptoms, *n* (%)**	
Fever	159 (100)
Myalgia	83 (52.2)
Headache	78 (49.1)
Arthralgia	70 (44)
Rash	60 (37.7)
Diarrhea	45 (28.3)
Nausea	35 (22)
Vomiting	24 (15.1)
Bleeding	9 (5.7)
Abdominal pain	10 (6.3)
**Laboratory parameters, *n*/*n* performed (%)**	
Thrombocytopenia (NV: 150,000–350,000/µL)	84/156 (53.8)
Leukopenia (4000–11,000/µL)	100/156 (64.1)
ALT > 40 UI/L	80/154 (51.9)
LDH > 250 UI/L	71/118 (60.2)
CRP negative (<5 mg/L)	67/154 (43.5)
**WHO clinical classification**	
Dengue without warning signs	150 (94.3)
Dengue with warning signs	8 (5.0)
Severe dengue	1 (0.6)
**Inpatients, *n* (%)**	47 (29.6)
Hospitalization days, median (IQR)	4 (0–5)

NV, normal value; ALT, alanine aminotransferase; LDH, lactate dehydrogenase; CRP, C-reactive protein; WHO, World Health Organization; IQR, interquartile range.

**Table 3:** Confirmed cases based on laboratory criteria and list of laboratory tests performed.

	*N*	%
**Cases confirmed**		
By RT-PCR on plasma/urine	128	92.8
- Plasma only	56	43.8
- Urine only	10	7.8
- Plasma and urine	62	48.4
By serology	10	7.2
- Seroconversion	7	
- Neutralization test	3	
**Diagnostic analysis, positive/performed (%)**		
RT-PCR positive on plasma sample	118/151	78.1
RT-PCR positive on urine sample	72/125	57.6
RT-PCR positive on plasma + urine sample	62/147	42.2
Dengue IgM positive	109/153	71.2
Dengue IgG positive	66/151	43.7
Dengue IgM + IgG positive	54/153	35.3
Suspected secondary	50/114	43.9

RT-PCR, real-time polymerase chain reaction.

**Table 4:** Distribution of identified serotypes in our study according to the WHO regions visited.

Serotypes	Total, *n* (%)	America, *n* (%)	Southeast Asia, *n* (%)	Western Pacific, *n* (%)	Africa, *n* (%)	>1 WHO Region, *n* (%)
Serotype 1	37 (28.9)	19 (31.1)	14 (34.1)	3 (25)	0	1 (33.3)
Serotype 2	35 (27.3)	23 (37.7)	8 (19.5)	2 (16.7)	1 (50)	1 (33.3)
Serotype 3	31 (24.2)	16 (26.2)	10 (24.2)	4 (33.3)	1 (50)	0
Serotype 4	16 (12.5)	3 (4.9)	9 (22.0)	3 (25)	0	1 (33.3)
ND	9 (7.0)					

ND, not determined.

## Data Availability

The data presented in this study is available on request from the corresponding author due to privacy reasons.

## Supplementary Materials

The following supporting information can be downloaded at https://www.mdpi.com/article/10.3390/idr17050113/s1, Table S1: reported travel destinations of patients with dengue fever.

## References

[1] Paz-Bailey G., Adams L.E., Deen J., Anderson K.B., Katzelnick L.C. (2024). Dengue. Lancet.

[2] Holmes E.C. (1998). Molecular epidemiology and evolution of emerging infectious diseases. Br. Med. Bull..

[3] Cattaneo P., Salvador E., Manica M., Barzon L., Castilletti C., Di Gennaro F., Huits R., Merler S., Poletti P., Riccardo F. (2025). Transmission of autochthonous Aedes-borne arboviruses and related public health challenges in Europe 2007–2023: A systematic review and secondary analysis. Lancet Reg. Health-Eur..

[4] Souza-Neto J.A., Powell J.R., Bonizzoni M. (2019). *Aedes aegypti* vector competence studies: A review. Infect. Genet. Evol..

[5] Powell J.R. (2018). Mosquito-borne human viral diseases: Why *Aedes aegypti*?. Am. J. Trop. Med. Hyg..

[6] Santilli L., Canovari B., Balducci M., Corbelli G., Maracci M., Polenta A., Farinaccio Y., Ginevri F., Anzalone N., Franca L. (2025). Outbreak of autochthonous dengue in Fano, Pesaro-Urbino Province-Marche region, Italy, September 2024. Infection.

[7] Rovida F., Faccini M., Molina Granè C., Cassaniti L., Senatore S., Rossetti E., Scardina G., Piazza M., Campanini G., Lilleri D. (2025). Lombardy Dengue Network. The 2023 dengue outbreak in Lombardy, Italy: A one-health perspective. Travel Med. Infect. Dis..

[8] Vita S., Lalle E., Caputi P., Faraglia F., D’Abramo A., Bordi L., De Carli G., Sberna G., Giancola M.L., Maffongelli G. (2024). Dengue fever as autochthonous infectious disease in Italy: Epidemiological, clinical and virological characteristics. Travel Med. Infect. Dis..

[9] Barzon L., Gobbi F., Capelli G., Montarsi F., Martini S., Riccetti S., Sinigaglia A., Pacenti M., Pavan G., Rassu M. (2021). Autochthonous dengue outbreak in Italy 2020: Clinical, virological and entomological findings. J. Travel Med..

[10] Regional Offices. https://www.who.int/about/who-we-are/regional-offices.

[11] Lee M. (2017). Basic Skills in Interpreting Laboratory Data.

[12] WHO (2009). Dengue Guidelines for Diagnosis, Treatment, Prevention and Control. https://www.who.int/publications/i/item/9789241547871.

[13] Moschetta N., Raccagni A.R., Bianchi M., Diotallevi S., Lolatto R., Candela C., Uberti Foppa C., Gismondo M.R., Castagna A., Nozza S. (2023). Mpox neutralising antibodies at 6 months from mpox infection or MVA-BN vaccination: A comparative analysis. Lancet Infect. Dis..

[14] Factsheet for Health Professionals About Dengue. https://www.ecdc.europa.eu/en/dengue-fever/facts.

[15] Wilder-Smith A. (2021). Dengue during the COVID-19 pandemic. J. Travel Med..

[16] European Centre for Disease Prevention and Control (ECDC) (2024). Dengue. Annual Epidemiological Report for 2022.

[17] Duvignaud A., Stoney R.J., Angelo K.M., Chen L.H., Cattaneo P., Motta L., Gobbi F.G., Bottieau E., Bourque D.L., Popescu C.P. (2024). Epidemiology of travel-associated dengue from 2007 to 2022: A GeoSentinel analysis. J. Travel Med..

[18] Calleri G., Torta I., Gobbi F., Angheben A., Lipani F., Lucchini A., Burdino F., Ghisetti V., Caramello P. (2017). Imported dengue in two tertiary Italian hospitals: Use of rapid diagnostic tests. Bull. Soc. Pathol. Exot..

[19] Tavakolipoor P., Schmidt-Chanasit J., Burchard G.D., Jordan S. (2016). Clinical features and laboratory findings of dengue fever in German travellers: A single-centre, retrospective analysis. Travel Med. Infect. Dis..

[20] Chen L.H., Marti C., Diaz Perez C., Jackson B.M., Simon A.M., Lu M. (2023). Epidemiology and burden of dengue fever in the United States: A systematic review. J. Travel Med..

[21] Huits R., Angelo K.M., Amatya B., Barkati S., Barnett E.D., Bottieau E., Emetulu H., Epelboin L., Eperon G., Medebb L. (2023). Clinical Characteristics and Outcomes Among Travelers With Severe Dengue: A GeoSentinel Analysis. Ann. Intern. Med..

[22] Trojanek M., Maixner J., Sojkova N. (2016). Dengue fever in Czech travellers: A 10-year retrospective study in a tertiary care centre. Travel Med. Infect. Dis..

[23] Ahmed A.M., Mohammed A.T., Vu T.T., Khattab M., Doheim M.F., Mohamed A.A., Abdelhamed M.M., Shamandy B.E., Daod M.T., Alesai W.A. (2020). Prevalence and burden of dengue infection in Europe: A systematic review and meta-analysis. Rev. Med. Virol..

[24] Pagani G., Zanchetta N., Galimberti L., Oreni L., Passerini S., Giacomelli A., Cordier L., Gismondo M.R., Rizzardini G., Galli M. (2020). Imported dengue fever: A 16-years retrospective analysis in Milan (Italy) and a brief review of the European literature. Infez. Med..

[25] Cooper E.C., Ratnam I., Mohebbi M., Leder K. (2014). Laboratory features of common causes of fever in returned travelers. J. Travel Med..

[26] Sabrina R.S.A., Azami N.A.M., Yap W.B. (2025). Dengue and Flavivirus Co-Infections: Challenges in Diagnosis, Treatment, and Disease Management. Intern. J. Mol. Sci..

[27] Majumdar A., Gupta R., Chatterjee A., Banu H., Biswas M., Gupta R., Mukherjee S., Sadhukhan P., Dutta S. (2023). A retrospective analysis of serological & molecular testing data on dengue fever in Kolkata & adjacent districts during 2016–2019. Indian J. Med. Res..

[28] Chan K.R., Ismail A.A., Thergarajan G., Raju C.S., Yam H.C., Rishya M., Sekaran S.D. (2022). Serological cross-reactivity among common flavivirus. Front. Cell. Infect. Microbiol..

[29] Roßbacher L., Malafa S., Huber K., Thaler M., Aberle S.W., Aberle J.H., Heinz F.X., Stiasny K. (2023). Effect of previous heterologous flavivirus vaccinations on human antibody responses in tick-borne encephalitis and dengue virus infections. J. Med. Virol..

[30] Maeki T., Tajima S., Ando N., Wakimoto Y., Hayakawa K., Kutsuna S., Kato F., Taniguchi S., Nakayama E., Lim C.K. (2023). Analysis of cross-reactivity among flaviviruses using sera of patients with dengue showed the importance of neutralization tests with paired serum samples for the correct interpretations of serological test results for dengue. J. Infect. Chemother..

[31] Istituto Superiore di Sanità-Epicentro-Arbovirosi in Italia nel 2024. https://www.epicentro.iss.it/arbovirosi/dashboard-2024.

[32] Neumayr A., Munoz J., Schunk M., Cramer J., Calleri G., Lopez-Velez R., Angheben A., Zoller T., Visser L., Serre-Delcor N. (2017). Sentinel surveillance of imported dengue via travellers to Europe 2012 to 2014: TropNet data from the DengueTools Research Initiative. Eurosurveillance.

[33] Lachmann R., Frank C., Wilking H., Kling K. (2025). Dengue virus infection in travellers after dengue vaccination, Germany 2023–24. J. Travel Med..

