# Supplementary material for: Imported Dengue Fever in Milan, Italy: A Seven-Year Retrospective Study
Source: Infect Dis Rep. 2025 Sep 12;17(5):113. doi: 10.3390/idr17050113 (PMC12452528; doi:10.3390/idr17050113)
Supplement: Supplementary file 1 [file idr-17-00113-s001.zip › idr-3733056-supplementary.pdf]

Table S1. Reported travel destinations of patients with Dengue Fever

Destination	*N*	%
**Africa**	**9**	**5.7**
Gambia + Mauritania + Algeria + Tunisia	1	0.6
Kenya	1	0.6
Madagascar + Mauritius	1	0.6
Mauritius (+ UAE)	1	0.6
Nigeria	2	1.3
Senegal	2	1.3
Tanzania	1	0.6
**America**	**75**	**47.2**
Argentina	3	1.9
Bolivia	1	0.6
Brazil	7	4.4
Caribbean (French Antilles)	4	2.5
Colombia	2	1.3
Costa Rica	5	3.1
Cuba	27	17.0
Cuba + Dominican Republic	1	0.6
Dominican Republic	2	1.3
Ecuador	1	0.6
El Salvador	1	0.6
Jamaica	2	1.3
Guatemala	2	1.3
Guatemala+ Belize	1	0.6
Haiti	2	1.3
Mexico	8	5.0
Mexico + Belize	1	0.6
Perù	4	2.5
Perù + Chile + (Polynesia)	1	0.6
**South East Asia**	**57**	**35.8**
India	4	2.5
India + Bhutan	1	0.6

India + Nepal	1	0.6
India + Thailand + Singapore + Philippines	1	0.6
Indonesia	9	5.7
Maldives	13	8.2
Maldives + Sri Lanka	1	0.6
Myanmar	1	0.6
Nepal	4	2.5
Sri Lanka	2	1.3
Thailand	18	11.3
Thailand (+ Cambodia)	2	1.3
**Western Pacific**	**18**	**11.3**
Cambodia	3	1.9
Cambodia + Vietnam	2	1.3
Laos	1	0.6
Philippines	4	2.5
Polynesia	4	2.5
Polynesia + China	1	0.6
Republic of Palau	1	0.6
Thailand + Myanmar + Laos + Cambodia + Vietnam + China + South Korea +Philippines + Malaysia + Indonesia	1	0.6
